# Supplementary material for: Scalable and programmable topological transitions in plasmonic Moiré superlattices
Source: Nat Commun. 2026 Jan 22;17:1931. doi: 10.1038/s41467-026-68635-6 (PMC12923820; doi:10.1038/s41467-026-68635-6)
Supplement: Supplementary file 1 — Supplementary Information [file 41467_2026_68635_MOESM1_ESM.pdf]

Supplementary Information

Scalable and Programmable Topological Transitions in Plasmonic Moiré Superlattices

Bo Tian[1], Xi Zhang[2], Ruitao Wu[1], Yuquan Zhang[1], Luping Du[1*], Xiaocong Yuan[1]

[1]Nanophotonics Research Centre, Shenzhen Key Laboratory of Micro-Scale Optical Information Technology, Institute of Microscale Optoelectronics & State Key Laboratory of Radio Frequency Heterogeneous Integration, Shenzhen University, Shenzhen 518060, China

[2]Guangdong Provincial Key Laboratory of Micro/Nano Optomechatronics Engineering, College of Mechatronics and Control Engineering, Shenzhen University, Shenzhen 518060, China

*Corresponding author. Email: lpdu@szu.edu.cn

Supplementary Note 1. Analysis of electric fields formed by the interference of six TM-polarized evanescent plane waves

In this section, we analyze the vector field generated by the interference of six TM-mode evanescent waves with respect to the phase. This goal is to show how phase variations can modulate the topological characteristics of the resulting vector field. Specifically, we first derive the mathematical expression describing the six TM-mode evanescent wave interference. Subsequently, to conveniently and comprehensively describe the effect of the phase, we define a phase parameter β that comprehensively encapsulates all possible phase variations. Finally, we present the evolution of the interference vector field as a function of this phase parameter.

Considering a time-harmonic monochromatic TM-polarized evanescent plane wave in a source-free, homogeneous and isotropic medium, its out-of-plane electric field component E_z can be described in the cylindrical coordinate $(\boldsymbol{r}, z)$[1]:

$$E_z(\mathbf{r}, z, t) = E_0' e^{-k_z z} e^{i(\mathbf{k_s}\cdot\mathbf{r}-\omega t+\varphi)}, \tag{S1}$$

where E_0' denotes the amplitude, $\mathbf{k_s}$ is the in-plane wavevector, k_z is the attenuation coefficient in the z-direction and φ is the initial phase.

Next, considering two of such evanescent waves are propagating in the opposite directions, they will interfere and generate a standing wave. In this case, the resulting electric field E_z is given by:

$$\begin{aligned} E_z(\mathbf{r}, z, t) &= E_0' \mathrm{e}^{-k_z \mathrm{z}}\left[\mathrm{e}^{\mathrm{i}(\mathbf{k_s}\cdot\mathbf{r}-\omega t+\varphi)} + \mathrm{e}^{\mathrm{i}(-\mathbf{k_s}\cdot\mathbf{r}-\omega t-\varphi)}\right] \\ &= E_0 e^{-k_z z} e^{-\mathrm{i}\omega t}\cos(\mathbf{k_s}\cdot\mathbf{r}+\varphi), \end{aligned} \tag{S2}$$

where E_0=2E_0'.

For the hexagonal structure discussed in the main text, we shall consider the case of the interference among three pairs of these standing waves. Without the loss of generosity, we assume that all three standing waves have identical amplitudes. Each of these standing waves is described in Eq. (S2). The directions of the wave vectors differ sequentially by $\pi/3$. Additionally, we shall neglect the time-varying phase term. Finally, the associated E_z component can be expressed as:

$$E_z(x, y, z) = E_0 e^{-k_z z} \sum_{\alpha=1}^{3} \cos\left(\mathbf{k}_{\mathbf{s},\alpha}\cdot\mathbf{r}+\varphi_\alpha\right), \tag{S3}$$

where $\mathbf{k}_{\mathbf{s},\alpha}\cdot\mathbf{r} = k_s x\cos(\phi_\alpha) + k_s y\sin(\phi_\alpha)$, ϕ_α denotes the direction of the in-plane wave vector $\mathbf{k}_{\mathbf{s},\alpha}$ and is sequentially different by $\pi/3$, $k_s = \left|\mathbf{k}_{\mathbf{s},\alpha}\right|$, φ_α represents the phase of three pairs of standing waves.

One might notice that the E_z component of vector fields described by Eq. (S3) has hexagonal lattice structures. Besides, the z-component of the electric field E_z is

associated with the x- (E_x) and y-component (E_y) by the following equations[1]:

$$E_x = -\frac{k_z}{k_s^2}\frac{\partial E_z}{\partial x}, \text{ and } E_y = -\frac{k_z}{k_s^2}\frac{\partial E_z}{\partial y}. \tag{S4}$$

From the above discussion, we can further evaluate the x- and y-component of the electric field by substituting Eq. (S3) into Eq. (S4):

$$E_x(x,y,z) = E_0 e^{-k_z z}\sum_{\alpha=1}^{3}\frac{k_z}{k_s}\cos(\phi_\alpha)\sin\left(\mathbf{k}_{\mathrm{s},\alpha}\cdot\mathbf{r}+\varphi_\alpha\right), \tag{S5a}$$

$$E_y(x,y,z) = E_0 e^{-k_z z}\sum_{\alpha=1}^{3}\frac{k_z}{k_s}\sin(\phi_\alpha)\sin\left(\mathbf{k}_{\mathrm{s},\alpha}\cdot\mathbf{r}+\varphi_\alpha\right). \tag{S5b}$$

We would like to emphasize that, the combination of both Eqs. (S3) and (S5) describes both the direction and components of the electric field within the hexagonal optical structures.

For the two-wave standing wave described in Eq. (S2), the variation of the phase φ will only result in a spatial translation of the superposed vector field, while preserving its global structure. However, for the six-wave interference pattern characterized by Eq. (S3), adjusting the phases φ_1, φ_2 and φ_3 will alter the global configuration, which will further lead to the change of the topological properties of the electromagnetic vector field.

To more conveniently characterize the modulation of the topological state of the vector field by the phase, we introduce a phase parameter β:

$$\beta = \varphi_2 - \varphi_1 - \varphi_3, \tag{S6}$$

which encapsulates all possible combinations of $(\varphi_1, \varphi_2, \varphi_3)$ and significantly reduces the complexity of the problem. It can be demonstrated that different combinations of $(\varphi_1, \varphi_2, \varphi_3)$ with the same β value result in a translationally shifted form of an identical vector field. Therefore, the topology of this hexagonal optical system can be explored by continuously modulating β.

To verify this, consider any two sets of parameters $(\varphi_1', \varphi_2', \varphi_3')$ and $(\varphi_1, \varphi_2, \varphi_3)$ that satisfy $\varphi_2 - \varphi_1 - \varphi_3 = \varphi_2' - \varphi_1' - \varphi_3'$. When Eq. (S3) is reformulated by defining a vector $\Delta\mathbf{r} = \frac{\Delta\varphi_1\hat{\mathbf{k}}_{\mathbf{s},\mathbf{1}} + \frac{-\sqrt{3}\Delta\varphi_1 + 2\sqrt{3}\Delta\varphi_2}{3}\hat{\mathbf{k}}_{\mathbf{s},\mathbf{1}}'}{k_s}$, the equation becomes:

$$
\begin{aligned}
E_z &= E_0 e^{-k_z z} \sum_{\alpha=1}^{3} \cos\left[\mathbf{k}_{s,\alpha} \cdot (\mathbf{r} + \Delta\mathbf{r}) + \varphi_\alpha\right] \\
&= E_0 e^{-k_z z} \left\{\cos\left[\mathbf{k}_{s,1} \cdot \mathbf{r} + \hat{\mathbf{k}}_{s,1} \cdot \left(\Delta\varphi_1 \hat{\mathbf{k}}_{s,1} + \frac{-\sqrt{3}\Delta\varphi_1 + 2\sqrt{3}\Delta\varphi_2}{3} \hat{\mathbf{k}}'_{s,1}\right) + \varphi_1\right]\right. \\
&\quad + \cos\left[\mathbf{k}_{s,2} \cdot \mathbf{r} + \hat{\mathbf{k}}_{s,2} \cdot \left(\Delta\varphi_1 \hat{\mathbf{k}}_{s,1} + \frac{-\sqrt{3}\Delta\varphi_1 + 2\sqrt{3}\Delta\varphi_2}{3} \hat{\mathbf{k}}'_{s,1}\right) + \varphi_2\right] \\
&\quad \left. + \cos\left[\mathbf{k}_{s,3} \cdot \mathbf{r} + \hat{\mathbf{k}}_{s,3} \cdot \left(\Delta\varphi_1 \hat{\mathbf{k}}_{s,1} + \frac{-\sqrt{3}\Delta\varphi_1 + 2\sqrt{3}\Delta\varphi_2}{3} \hat{\mathbf{k}}'_{s,1}\right) + \varphi_3\right]\right\} \\
&= E_0 e^{-k_z z} \left\{\cos\left[\mathbf{k}_{s,1} \cdot \mathbf{r} + \Delta\varphi_1 + \varphi_1\right]\right. \\
&\quad + \cos\left[\mathbf{k}_{s,2} \cdot \mathbf{r} + \left(\frac{1}{2}\Delta\varphi_1 + \frac{\sqrt{3}}{2} \frac{-\sqrt{3}\Delta\varphi_1 + 2\sqrt{3}\Delta\varphi_2}{3}\right) + \varphi_2\right] \\
&\quad \left. + \cos\left[\mathbf{k}_{s,3} \cdot \mathbf{r} + \left(-\frac{1}{2}\Delta\varphi_1 + \frac{\sqrt{3}}{2} \frac{-\sqrt{3}\Delta\varphi_1 + 2\sqrt{3}\Delta\varphi_2}{3}\right) + \varphi_3\right]\right\} \\
&= E_0 e^{-k_z z} \sum_{\alpha=1}^{3} \cos\left(\mathbf{k}_{s,\alpha} \cdot \mathbf{r} + \varphi'_\alpha\right),
\end{aligned} \tag{S7}
$$

where $\Delta\varphi_1 = \varphi'_1 - \varphi_1$, $\Delta\varphi_2 = \varphi'_2 - \varphi_2$, $\hat{\mathbf{k}}_{s,\alpha}$ denotes the unit vector in the $\mathbf{k}_{s,\alpha}$ direction, and $\hat{\mathbf{k}}'_{s,1}$ denotes the unit vector perpendicular to $\hat{\mathbf{k}}_{s,1}$. Eq. (S7) indicates that the parameter β uniquely determines the topological configuration of the vector field described by Eq. (S3).

In this study, we choose the phase values $\varphi_2 = \beta/3$, $\varphi_1 = -\beta/3$ and $\varphi_3 = -\beta/3$ to construct and investigate the topology of the electric field described in Eq. (S3) and (S5). This choice of phase distribution ensures that the center of the unit cell is always located at the origin of the coordinates, allowing us to more conveniently explore how the continuous modulation of β influences the topological properties of the field. Supplementary Fig. 1 illustrates how the E_z component changes as β varies. The figure reveals that the vector field consistently maintains a hexagonal lattice structure outlined by the black dashed line. However, the distribution of the vector field within this structure exhibits periodic variations with a period of 2π as β changes. The topological invariants (TIs) of these vector fields are calculated using Eq. (1) from the main text, with the results presented in Fig. 1d of the main text. Specifically, the TIs take the following values: 1 for Supplementary Fig. 1(a-c, k, l), 0 for Supplementary Fig. 1(d, j), -1 for Supplementary Fig. 1(e-i). These values indicate different topological states of the vector field as β is modulated, with the transitions between these states reflecting changes in the field's topological configuration.

Supplementary Note 2. The mechanism for the topological transitions

In this section, we describe the mechanisms underlying topological transitions (TTs). Our analysis is structured into two parts. First, we investigate the evolution of band structures in topological insulators and systematically analyze the topological phase transition conditions of Chern numbers within the two-band model, establishing that topological phase transition stems from singularities emerging in the modulation-induced vector field. Second, through the definition of skyrmion, we demonstrate how singularities directly induce mutation of TIs of skyrmion, thereby revealing the essential connection between vector field singularities and transformations in topological properties.

In topological insulators, the variation of the energy band structures is shown in Supplementary Fig. 8 when a topological phase transitions occurs[2]. This characterization of the energy bands can be quantitatively analyzed by the two-band model. In the two-band model, the Hamiltonian can be expressed as[3]:

$$\widehat{\mathbf{H}}(\mathbf{h}) = h_x\hat{\sigma}_x + h_y\hat{\sigma}_y + h_z\hat{\sigma}_z = \begin{bmatrix} h_z & h_x - \mathrm{i}h_y \\ h_x + \mathrm{i}h_y & -h_z \end{bmatrix}, \tag{S8}$$

where $\mathbf{h} = (h_x, h_y, h_z)$ is related to the hopping strengths, and $\hat{\boldsymbol{\sigma}} = (\hat{\sigma}_x, \hat{\sigma}_y, \hat{\sigma}_z)$ represents the Pauli matrices:

$$\hat{\sigma}_x = \begin{bmatrix} 0 & 1 \\ 1 & 0 \end{bmatrix},\ \hat{\sigma}_y = \begin{bmatrix} 0 & -\mathrm{i} \\ \mathrm{i} & 0 \end{bmatrix},\ \hat{\sigma}_z = \begin{bmatrix} 1 & 0 \\ 0 & -1 \end{bmatrix}. \tag{S9}$$

The energy bands can be derived by solving the eigenvalues $\varepsilon(\mathbf{k})$ of the matrix described in Eq. (S8). Solving for $\varepsilon(\mathbf{k})$ leads to the following determinant condition:

$$\begin{vmatrix} h_z - \varepsilon(\mathbf{k}) & h_x - \mathrm{i}h_y \\ h_x + \mathrm{i}h_y & -h_z - \varepsilon(\mathbf{k}) \end{vmatrix} = 0, \tag{S10}$$

and the form of $\varepsilon(\mathbf{k})$ can be derived to be:

$$\varepsilon(\mathbf{k}) = \pm|\mathbf{h}| = \pm\sqrt{h_x^2 + h_y^2 + h_z^2}, \tag{S11}$$

where $\sqrt{h_x^2 + h_y^2 + h_z^2}$ and $-\sqrt{h_x^2 + h_y^2 + h_z^2}$ corresponding to the upper and lower energy bands in Supplementary Fig. 8, respectively. The two bands in the model physically represent the valence and conduction bands, whose interplay through band inversion and Berry curvature determines the system's topological properties.

The topological properties of the energy bands are characterized by the Chern Number (CN). The general form of the CN is given by[3]:

$$\mathrm{CN} = \frac{\mathbf{1}}{\mathbf{4\pi}} \int_{\mathrm{FBZ}} \nabla_{\mathbf{k}} \times \langle u_n(\mathbf{k}) | \boldsymbol{i}\nabla_{\mathbf{k}} | u_n(\mathbf{k}) \rangle \, d\mathbf{k}, \tag{S12}$$

where $u_n(\mathbf{k})$ are the eigenstates, FBZ denotes first Brillouin zone, $\boldsymbol{\nabla}_{\mathbf{k}}$ is a differential operator in momentum space. For the two-band model described by Eqs. (S8-S11), the CN can be written as[3]:

$$\mathrm{CN} = \frac{\mathbf{1}}{\mathbf{4}\boldsymbol{\pi}}\int_{FBZ} \mathbf{h}\cdot\left(\partial_{k_x}\mathbf{h}\times\partial_{k_y}\mathbf{h}\right)d\mathbf{k}. \tag{S13}$$

The CN quantifies the topological properties of band structures and a non-zero CN indicates a non-trivial band.

From Eq. (S13) and Eq. (1) of the main text, the CN in the two-band model can indeed be interpreted as a TI of a skyrmion if we think of $\mathbf{h}$ as defining a mapping from the FBZ to the sphere S^2. Consequently, we can define a matrix $\widehat{\mathrm{M}}$ analogous to $\widehat{\mathrm{H}}$, taking the form $\widehat{\mathrm{M}} = \mathbf{E}\cdot\hat{\sigma}$, where $\mathbf{E} = \left(E_x, E_y, E_z\right)$. The eigenvalues for $\widehat{\mathrm{M}}$ are therefore: $\mathrm{M}_\pm=\pm|\mathbf{E}|= \pm\sqrt{E_x^2+E_y^2+E_z^2}$, allowing for the construction of an energy band-like structure in real space. In the two-band model, the band structure evolution during the topological phase transition follows the process illustrated in Supplementary Fig. 8, with a singularity emerging at the band closure. Similarly, for the vector fields governed by Eqs. (S3) and (S5), the energy band-like structures $\mathrm{M}_\pm$ undergo gap closing and reopening, accompanied by singularities in the vector field at the closure point. This behavior is directly observed in Figs. 1e and 1f of the main text, where the phase parameters transition clearly shows the band-like gap closing and reopening, alongside the concurrent appearance of a vector field singularity.

Next, we will demonstrate that singularities lead to mutations of TIs by means of the definition of the skyrmion. The TI of skyrmion counts the number of times a vector field wraps the unit sphere. Therefore, a mutation in the TI means that there is a mutation in the degree to which the vector directions cover the unit sphere. For Eqs. (S3) and (S5), there are singularities at the positions marked by the red dots in Supplementary Fig. 1d when $\beta=\pi/2$, and their coordinates are (0, $2\lambda/3$), ($-\sqrt{3}\lambda/3$, $-\lambda/3$), and ($\sqrt{3}\lambda/3$, $-\lambda/3$). We illustrate why the generation of singularities leads to a mutation of TI by using the singularity with the coordinate (0, $2\lambda/3$).

According to Eqs. (S3) and (S5), when x=0, $y\in[0, 2\lambda/3]$

$$E_x(0,y) = 0, \tag{S14a}$$

$$E_y(0,y) = 2E_0\frac{k_z}{k_s}\sin\left(\frac{3k_sy}{4}\right)\cos\left(\frac{k_sy}{4}+\frac{\beta}{3}\right), \tag{S14b}$$

$$E_z(0,y) = E_0\left[2\cos\left(\frac{k_sy}{2}-\frac{\beta}{3}\right)+\cos\left(k_sy+\frac{\beta}{3}\right)\right], \tag{S14c}$$

where ϕ_1, ϕ_2 and ϕ_3 are chosen as π/6, π/2 and 5π/6, respectively, for computational convenience. From Eq. (S14), the vector direction on the *y*-axis consistently lies within the *y*-*z* plane and can be determined by the ratio E_y/E_z:

$$\frac{E_y(0,y)}{E_z(0,y)}=\frac{2\frac{k_z}{k_s}\sin\left(\frac{3k_sy}{4}\right)\cos\left(\frac{k_sy}{4}+\frac{\beta}{3}\right)}{2\cos\left(\frac{k_sy}{2}-\frac{\beta}{3}\right)+\cos\left(k_sy+\frac{\beta}{3}\right)}. \tag{S15}$$

When $\beta\in[0,\pi/2)\cup(\pi/2,\pi]$, y tends to 2λ/3, we have

$$\frac{E_y(0,y)}{E_z(0,y)}=\frac{2\frac{k_z}{k_s}\sin\pi\cos\left(\frac{\pi}{3}+\frac{\beta}{3}\right)}{2\cos\left(\frac{2\pi}{3}-\frac{\beta}{3}\right)+\cos\left(\frac{4\pi}{3}+\frac{\beta}{3}\right)}=0. \tag{S16}$$

When β=π/2, y tends to $2\lambda/3$, we can obtain

$$\begin{aligned}\frac{E_y(0,y)}{E_z(0,y)}&=\frac{2\frac{k_z}{k_s}\sin\left(\frac{3k_sy}{4}\right)\cos\left(\frac{k_sy}{4}+\frac{\pi}{6}\right)}{2\cos\left(\frac{k_sy}{2}-\frac{\pi}{6}\right)+\cos\left(k_sy+\frac{\pi}{6}\right)}\\&=-\frac{k_z}{k_s}\frac{3\cos\left(\frac{3k_sy}{4}\right)\cos\left(\frac{k_sy}{4}+\frac{\pi}{6}\right)-\sin\left(\frac{3k_sy}{4}\right)\sin\left(\frac{k_sy}{4}+\frac{\pi}{6}\right)}{2\sin\left(\frac{k_sy}{2}-\frac{\pi}{6}\right)+2\sin\left(k_sy+\frac{\pi}{6}\right)}\\&=\frac{k_z}{k_s}\frac{5\cos\left(\frac{k_sy}{4}+\frac{\pi}{6}\right)\sin\left(\frac{3k_sy}{4}\right)+3\cos\left(\frac{3k_sy}{4}\right)\sin\left(\frac{k_sy}{4}+\frac{\pi}{6}\right)}{2\cos\left(\frac{k_sy}{2}-\frac{\pi}{6}\right)+4\cos\left(k_sy+\frac{\pi}{6}\right)}\\&=\infty.\end{aligned} \tag{S17}$$

Eqs. (S16) and (S17) show that the vector direction converges to the *z*-axis when $\beta\in[0,\pi/2)\cup(\pi/2,\pi]$ and to the *y*-axis when $\beta=\pi/2$. This mutation implies an abrupt change in the space occupied by the vector direction, which leads to an abrupt change in the TIs of the vector field. Similarly, a similar abrupt change occurs when $\beta=3\pi/2$.

While Eqs. (S16) and (S17) only show mutation in the *y*-axis when a singularity arises, the mutations actually occur in all directions. Supplementary Fig. 9 illustrates the vector direction in each direction near the singularity when the singularity arises and disappears. For convenience, the β are chosen as β=π/2-π/1e6 (Supplementary Fig. 9a), π/2 (Supplementary Fig. 9b) and π/2+π/1e6 (Supplementary Fig. 9c), respectively. As shown in the figure, when β=π/2, the center of the structure exhibits a singularity, with the surrounding vector field predominantly confined to the x-y plane. However, as β deviates from π/2, the singularity vanishes, and the vector's direction near the center tends to the *z*-axis. This indicates that even slight variations in β around the critical value of π/2 can induce an abrupt reorientation of the vector field.

In addition to this, we can find the change in the topological state of the vector field in Supplementary Fig. 9. At β=π/2-π/1e6, a bimeron with TI of 1 emerges. This structure undergoes complete annihilation precisely at β=pi/2. Strikingly, as β increases

to π/2+π/1e6, a new bimeron with TI of −1 nucleates. Consequently, the TI of the vector field decreases sequentially by 1 across Supplementary Figs. 9a to 9b to 9c, reflecting a TT in the system's topology. Note that Supplementary Figs. 9a-9c illustrate the change of the vector field in the case of β=π/2±π/1e6. In fact, this change occurs instantaneously when β deviates from π/2, which results in the TIs showing the quantization trend shown in Fig. 1d of the main text.

Supplementary Note 3. Optical Moiré superlattices

In this section, we present a methodology for generating optical Moiré superlattices and establishing control parameters to dynamically modulate their topological states.

As we mentioned in the main text, the Moiré superlattices can be formed by the interference of two lattices with the same structure but with an angle difference θ. The vector fields described by Eqs. (S3) and (S5) have hexagonal lattice structures, therefore an optical Moiré superlattice can be obtained by interfering with the vector fields described by Eqs. (S3) and (S5) after rotating it by a certain angle θ. The specific form can be expressed as follows:

$$E_z(x,y) = E_0 \sum_{\alpha=1}^{3}\left[\cos\left(\mathbf{k}_{\mathbf{s},\alpha,1}\cdot\mathbf{r}+\varphi_{\alpha,1}\right)+\cos\left(\mathbf{k}_{\mathbf{s},\alpha,2}\cdot\mathbf{r}+\varphi_{\alpha,2}\right)\right], \quad \text{(S18)}$$

where the subscripts 1 and 2 were introduced to distinguish the two hexagonal lattices and the x- (E_x) and y-component (E_y) can be derived using Eq (S4) and can be expressed as:

$$E_x = E_0 \sum_{\alpha=1}^{3}\frac{k_z}{k_s}\left[\cos\left(\phi_{\alpha,1}\right)\sin\left(\mathbf{k}_{\mathbf{s},\alpha,1}\cdot\mathbf{r}+\varphi_{\alpha,1}\right)+\cos\left(\phi_{\alpha,2}\right)\sin\left(\mathbf{k}_{\mathbf{s},\alpha,2}\cdot\mathbf{r}+\varphi_{\alpha,2}\right)\right], \quad \text{(S19a)}$$

$$E_y = E_0 \sum_{\alpha=1}^{3}\frac{k_z}{k_s}\left[\sin\left(\phi_{\alpha,1}\right)\sin\left(\mathbf{k}_{\mathbf{s},\alpha,1}\cdot\mathbf{r}+\varphi_{\alpha,1}\right)+\sin\left(\phi_{\alpha,2}\right)\sin\left(\mathbf{k}_{\mathbf{s},\alpha,2}\cdot\mathbf{r}+\varphi_{\alpha,2}\right)\right]. \quad \text{(S19b)}$$

Together, Eqs. (S18) and (S19) fully describe the direction and components of the electric field. For the vector field described by Eqs. (S18) and (S19), we introduce two phase parameters β_1 and β_2, which are related to the phase as:

$$\beta_1 = \varphi_{2,1} - \varphi_{1,1} - \varphi_{3,1}, \quad \text{(S20a)}$$

$$\beta_2 = \varphi_{2,2} - \varphi_{1,2} - \varphi_{3,2}. \quad \text{(S20b)}$$

We choose the phase values $\varphi_{2,1} = \beta_1/3$, $\varphi_{1,1} = -\beta_1/3$, $\varphi_{3,1} = -\beta_1/3$ and $\varphi_{2,2} = \beta_2/3$, $\varphi_{1,2} = -\beta_2/3$, $\varphi_{3,2} = -\beta_2/3$ to construct and investigate the topology of the electric field. Supplementary Fig. 2 illustrates how the E_z component described by Eq. (S18) changes as β_1 and β_1 vary. The figure reveals that the vector field consistently maintains a hexagonal superlattice structure outlined by the black dashed line. The TIs of these vector fields are calculated using Eq. (1) from the main text. These values indicate different topological states of the vector field as β_1 and β_1 are modulated, with the transitions between these states reflecting changes in the field's topological configuration.

Supplementary Note 4. The effect of singularities on the topological state

In this section, we analyze the TIs characterizing critical states during TTs, as well as the singularities that do not lead to TTs.

As shown in section 2, the singularity corresponds to a transient state of the TT. Notably, the magnitude of the TI change from Supplementary Fig. 9a to 9b is identical to that from Supplementary Fig. 9b to 9c, implying that the TI of the transient state (Supplementary Fig. 9b) is the arithmetic mean of the TIs in Supplementary Figs. 9a and 9c. This behavior extends to the system depicted in Fig. 2e of the main text, where transient states localize at boundaries between adjacent regions. Consequently, the TIs at these boundaries exactly match the average of the TIs in the neighboring regions.

Crucially, while singularities are necessary for TTs, they are not sufficient. This is clearly demonstrated in Fig. 2e of the main text: when the phase parameter follows the black dashed line, singularities emerge in the vector field, yet the TIs remain identical on both sides. The key determinant of TTs lies not merely in singularities formation, but rather in comparing the vector field's local structure before and after the singularity appears. Specifically, the topological state remains unchanged if the local configuration near the singularity is identical pre- and post-formation; conversely, any structural difference induces a TT.

To characterize the properties of the black dashed line more clearly, we analyze the trajectories of β_1 and β_2 as they cross one of these lines. Supplementary Fig. 10a depicts the evolution of β_1 and β_2, where the trajectories follow the diagonal (β_1=β_2), with the starting point "a", ending point "c", and intersection point "b". For clarity, we denote the phase parameters at these points as β_a, β_b, and β_c, respectively. Supplementary Fig. 10b shows the vector field modes when β_1=β_2=β_b, with three singularities highlighted by black circles. To further elucidate the influence of singularities on the vector field's topological state, we examine the vector orientations near the singularities at β_1=β_2=β_a (Supplementary Fig. 10c), β_1=β_2=β_b (Supplementary Fig. 10d) and β_1=β_2=β_c (Supplementary Fig. 10e), respectively. A key observation is that an antimeron with identical structure and TI equal to 0.5 emerges near the singularity at both β_1=β_2=β_a (Supplementary Fig. 10c) and β_1=β_2=β_c (Supplementary Fig. 10e), but vanishes at β_b (Supplementary Fig. 10d). This disappearance and subsequent reappearance of the antimeron ensures that the structures in Supplementary Figs. 10c and 10e are identical, confirming that the vector field's topological state remains unchanged.

Last, we should note that, the distances between points "a", "b", and "c" are

enlarged in Supplementary Fig. 10a for clear illustration. In the actual calculation, we have $\beta_a=\beta_b-\pi/1e6$, $\beta_c=\beta_b+\pi/1e6$, $\beta_b\approx0.89\pi$. In other words, they are very close to each other.

Supplementary Note 5. Moiré angle dependent topological invariants

The size of unit cells of the Moiré superlattices varies with the Moiré angle, which leads to the range of TIs varying with the Moiré angle. As shown in Fig. 2e of the main text, the TIs for the case (m, n)=(2, 3) span all integers and half-integers from −8 to 8, where zeros and half-integers lie along the boundaries of the small regions in the phase diagram. This range expands significantly with varying Moiré angles—for instance, it increases from −19 to 19 at (m, n)=(3, 4) and further extends from −58 to 58 at (m, n)=(4,9).

The phase parameters β_1 and β_2 corresponding to different TIs are summarized in Supplementary Tables 1 and 2. Notably, for non-zero integer invariants, the phase parameters can vary continuously within a region; the values listed in the tables represent only one possible configuration within these regions. Consequently, the TI remains stable under small variations of β_1 and β_2 around the tabulated values.

In contrast, when the TI equals zero, the phase parameters reside precisely on the boundaries of the small regions in Fig. 2e. Any deviation from β_1=β_2=1.5π results in an abrupt change in the TI. For half-integer TIs, the exact phase parameters at these boundaries cannot be expressed as specific numerical values, which is why only integer-invariant cases are included in Supplementary Tables 1 and 2.

Supplementary Note 6. The properties of singularities

As shown in Section 2, the properties of singularities play a critical role in determining the topological properties of the vector field after a TT. Notably, these properties are not universal—they vary across different singularities, meaning the topological state depends not only on the number of singularities but also on their intrinsic characteristics.

To illustrate this, consider the singularity structure depicted in Supplementary Fig. 9b. Our analysis reveals that when the system evolves from the configuration in Supplementary Fig. 9a to the structure in Supplementary Fig 9b and finally to Supplementary Fig. 9c, the TI changes by 2. Conversely, the reverse transition (9c → 9b → 9a) yields a TI change of −2. This demonstrates that for the specific singularity in Supplementary Fig. 9b, the possible TI changes are strictly ±2, where the sign is determined by the transition direction. The magnitude of the TI change is directly tied to the winding number of the vector field around the singularity. Therefore, when multiple singularities emerge during a TT, the total change in the TI equals the sum of the winding numbers associated with all singularities.

Supplementary Note 7. Multilayer optical hexagonal Moiré superlattice

In Section 3, the bilayer hexagonal Moiré superlattice is introduced. When the number of layers increases to P layers, the expression for the vector field is given by:

$$E_z = E_0 \sum_{a=1}^{P} \sum_{\alpha=1}^{3} \cos(\mathbf{k}_{\mathbf{s},\alpha,\mathrm{a}} \cdot \mathbf{r} + \varphi_{\alpha,\mathrm{a}}), \tag{S21a}$$

$$E_x = E_0 \sum_{a=1}^{P} \sum_{\alpha=1}^{3} \frac{k_z}{k_s} \cos(\phi_{\alpha,\mathrm{a}}) \sin(\mathbf{k}_{\mathbf{s},\alpha,\mathrm{a}} \cdot \mathbf{r} + \varphi_{\alpha,\mathrm{a}}), \tag{S21b}$$

$$E_y = E_0 \sum_{a=1}^{P} \sum_{\alpha=1}^{3} \frac{k_z}{k_s} \sin(\phi_{\alpha,\mathrm{a}}) \sin(\mathbf{k}_{\mathbf{s},\alpha,\mathrm{a}} \cdot \mathbf{r} + \varphi_{\alpha,\mathrm{a}}), \tag{S21c}$$

where $\mathbf{k}_{\mathbf{s},\alpha,\mathrm{a}} \cdot \mathbf{r} = k_s x\cos(\phi_{\alpha,\mathrm{a}}) + k_s y\sin(\phi_{\alpha,\mathrm{a}})$, $\phi_{\alpha,\mathrm{a}}$ denotes the direction of the in-plane wave vector $\mathbf{k}_{\mathbf{s},\alpha,\mathrm{a}}$, $k_s = |\mathbf{k}_{\mathbf{s},\alpha,\mathrm{a}}|$, $\varphi_{\alpha,\mathrm{a}}$ represents the phase. For a P-layer hexagonal Moiré superlattice, the number of phase parameters β_a to be introduced is P. The relationship between each phase parameter and the phase can be expressed as:

$$\beta_\mathrm{a} = \varphi_{2,\mathrm{a}} - \varphi_{1,\mathrm{a}} - \varphi_{3,\mathrm{a}}. \tag{S22}$$

In our calculations, we assign the phase values $\varphi_{2,\mathrm{a}} = \beta_a/3$, $\varphi_{1,\mathrm{a}} = -\beta_a/3$ and $\varphi_{3,\mathrm{a}} = -\beta_a/3$ to construct and investigate the topology of the electric field. Supplementary Figs. 11a-p illustrate the evolution of the E_z component described by Eq. (S21a) as β_1, β_2 and β_3 are varied simultaneously for P=3, at a Moiré angle of 21.8 degrees. These panels show that although the vector field distribution grows increasingly complex, the topological invariants still never take on values that are integer multiples of 3/2. This occurs because, although structural complexity increases with additional layers, the underlying symmetry remains unchanged, and hence the selection rule governing topological states persists. Supplementary Figs. 11q and 11r show the front views of the energy band-like structures corresponding to the three-layer hexagonal Moiré superlattices. As the structures become more complex, the resulting band-like structures also become increasingly intricate; nonetheless, clear features such as band gaps (Fig. 11q) and band gap closure (Fig. 11r) remain observable.

Supplementary Note 8. Optical square Moiré superlattice

The existence rule of topological invariants is closely tied to structural symmetry. When the structural symmetry changes, the criteria for the existence of topological states must be reexamined. We illustrate this using a square Moiré superlattice as an example. The vector field of a P-layer square Moiré superlattice can be expressed as:

$$E_z = E_0 \sum_{a=1}^{P} \sum_{\alpha=1}^{2} \cos(\mathbf{k}_{\mathbf{s},\alpha,\mathrm{a}} \cdot \mathbf{r} + \varphi_{\alpha,\mathrm{a}}), \quad \text{(S23a)}$$

$$E_x = E_0 \sum_{a=1}^{P} \sum_{\alpha=1}^{2} \frac{k_z}{k_s} \cos(\phi_{\alpha,\mathrm{a}}) \sin(\mathbf{k}_{\mathbf{s},\alpha,\mathrm{a}} \cdot \mathbf{r} + \varphi_{\alpha,\mathrm{a}}), \quad \text{(S23b)}$$

$$E_y = E_0 \sum_{a=1}^{P} \sum_{\alpha=1}^{2} \frac{k_z}{k_s} \sin(\phi_{\alpha,\mathrm{a}}) \sin(\mathbf{k}_{\mathbf{s},\alpha,\mathrm{a}} \cdot \mathbf{r} + \varphi_{\alpha,\mathrm{a}}), \quad \text{(S23c)}$$

where $\phi_{\alpha,\mathrm{a}}$ is sequentially different by $\pi/2$. Supplementary Fig. 12 illustrates the evolution of the vector field in the square Moiré superlattice. Supplementary Fig. S12a displays the distribution of the vector field in the square lattice for the case of P=1. In this configuration, arbitrary variations in the phase φ_α do not alter the structure of the square lattice. Consequently, for a single-layer square lattice, the vector field consistently maintains a topological invariant of 0, as depicted in Supplementary Fig. 12a.

Supplementary Figs. 12b-j demonstrate the evolution of the vector field described by Eq. (S23a) for P=2. Since the structure of the vector field is immune to phase variations in the P=1 case, the phase $\varphi_{1,1}$ and $\varphi_{1,2}$ can be held fixed in the bilayer structure, while only $\varphi_{2,1}$ and $\varphi_{2,2}$ are varied. These figures track the resulting changes in the vector field. The resulting structures are observed to lack C_3 symmetry, and calculations show that the topological invariant remains 0 throughout this process, indicating no change in the topological state. This can only be due to one of two reasons: first, the energy band-like structure neither closes nor reopens; second, although band closing and reopening occur, this process fails to induce a topological transition (as energy band-like closing and reopening are necessary but not sufficient conditions for topological transition). Supplementary Figs. 12k and 12l show the energy band-like when $\varphi_{2,1}$ and $\varphi_{2,2}$ are 0 and 1, respectively. One structure exhibits a bandgap, while the other is gapless, confirming that the energy band-like structure indeed undergoes closing and reopening during the phase variation. Thus, the absence of a topological transition must be attributed to the second reason.

Section 5 has already demonstrated cases where band closing and reopening occur without a change in the topological invariant. For the square Moiré superlattice, however, the underlying mechanism differs. The mechanism is elucidated by examining the singularity in the vector field when $\varphi_{2,1}$ and $\varphi_{2,2}$ equal π. In Supplementary Fig. 12f, singularies of different types within the unit cell structure are marked with red, black, green, and blue points. Since singularity on the unit cell boundary are shared by multiple unit cells, the unit cell in Supplementary Fig. 12f contains only one of each type of singularity. Supplementary Figs. 12m-p display the distribution of the vector field near the red, black, blue, and green singularities, respectively. It is evident that the winding numbers near the red and black singularities are opposite, as are those near the blue and green singularities. This indicates that the effects of these singularities cancel each other out, resulting in a net contribution of zero. The change in the topological invariant after a topological transition depends on the number and nature of singularities. Therefore, the topological state of the vector field in the square Moiré superlattice remains unchanged during phase modulation due to the pairwise emergence of singularities with opposite winding numbers.

Supplementary Note 9. Optical topological states with losses

Section 7 does not account for system losses. When losses are included, the vector fields forming the hexagonal Moiré superlattice remain consistent with the description in Eq. (S21). However, the in-plane wavenumber k_s becomes complex and can be expressed as $k_s = k_s' + ik_s''$. Substituting this complex wavenumber into Eq. (S21a) yields

$$
\begin{aligned}
E_z &= E_0 \sum_{a=1}^{P} \sum_{\alpha=1}^{3} \cos\left(\mathbf{k}_{\mathbf{s},\alpha,\mathrm{a}} \cdot \mathbf{r} + \varphi_{\alpha,\mathrm{a}}\right) \\
&= E_0 \sum_{a=1}^{P} \sum_{\alpha=1}^{3} \cos\left[k_s x \cos\phi_{\alpha,\mathrm{a}} + k_s y \sin\phi_{\alpha,\mathrm{a}} + \varphi_{\alpha,\mathrm{a}}\right] \\
&= E_0 \sum_{a=1}^{P} \sum_{\alpha=1}^{3} \cos\left[k_s' x \cos\phi_{\alpha,\mathrm{a}} + k_s' y \sin\phi_{\alpha,\mathrm{a}} + ik_s'' x \cos\phi_{\alpha,\mathrm{a}} + ik_s'' y \sin\phi_{\alpha,\mathrm{a}} + \varphi_{\alpha,\mathrm{a}}\right] \\
&= E_0 \sum_{a=1}^{P} \sum_{\alpha=1}^{3} \left\{\cos\left[k_s' x \cos\phi_{\alpha,\mathrm{a}} + k_s' y \sin\phi_{\alpha,\mathrm{a}} + \varphi_{\alpha,\mathrm{a}}\right] \cosh\left[k_s'' x \cos\phi_{\alpha,\mathrm{a}} + k_s'' y \sin\phi_{\alpha,\mathrm{a}}\right]\right. \\
&\qquad \left. - i \sin\left[k_s' x \cos\phi_{\alpha,\mathrm{a}} + k_s' y \sin\phi_{\alpha,\mathrm{a}} + \varphi_{\alpha,\mathrm{a}}\right] \sinh\left[k_s'' x \cos\phi_{\alpha,\mathrm{a}} + k_s'' y \sin\phi_{\alpha,\mathrm{a}}\right]\right\}.
\end{aligned}
\tag{S24}
$$

The z-component of the vector field in the TM-mode evanescent wave uniquely determines the x- and y-components through Eq. (S4), thereby fully characterizing the entire vector field. Eq. (S24) shows that the resulting electric field acquires an imaginary part. In previous work[4], a detailed analysis was conducted for the case where P=1 and $\phi_{\alpha,\mathrm{a}} = 0$ in Eq. (S24). It was shown that the skyrmion number is preserved provided that the real part of the field maintains the same functional form as Eq. (S21) and its magnitude is far larger than that of the imaginary part. We now demonstrate that this conclusion holds generally for Eq.(S24). Following the approach from previous work[4] for the case of P=1 and $\phi_{\alpha,\mathrm{a}} = 0$, we take $|k' - k_0| \gg |k''|$ and $x, y \ll 1/k''$. These two conditions imply, respectively, that the losses are sufficiently small and that the spatial extent under consideration is much smaller than the propagation distance of the electromagnetic wave. Under these two assumptions, a first-order Taylor series expansion of Eq. (S24) yields

$$
\begin{aligned}
E_z = E_0 \sum_{a=1}^{P} \sum_{\alpha=1}^{3} &\left\{\cos\left[k_s' x \cos\phi_{\alpha,\mathrm{a}} + k_s' y \sin\phi_{\alpha,\mathrm{a}} + \varphi_{\alpha,\mathrm{a}}\right]\right. \\
&\left. - i \sin\left[k_s' x \cos\phi_{\alpha,\mathrm{a}} + k_s' y \sin\phi_{\alpha,\mathrm{a}} + \varphi_{\alpha,\mathrm{a}}\right]\left[k_s'' x \cos\phi_{\alpha,\mathrm{a}} + k_s'' y \sin\phi_{\alpha,\mathrm{a}}\right]\right\}.
\end{aligned}
\tag{S25}
$$

The Eq. (S25) shows that its real part is identical to Eq. (S23a), while its imaginary part remains much smaller than the real part under the condition that $k_s'' x \ll 1$ and $k_s'' y \ll 1$. This result confirms that, when system losses are sufficiently small, the real part of the vector field remains a perfect platform for studying topological transitions.

Additionally, when system losses are taken into account, the translational symmetry of the hexagonal lattice is slightly broken. This results in a minor deviation

of the topological invariant of the vector field within the unit cell from quantized integer values. Nevertheless, for sufficiently small losses, the topological invariant derived from the real part of the vector field approaches integer values in well-defined topological states, in agreement with previous work[4]. This topological structure facilitates the experimental observation of transitions between distinct topological phases in real systems.

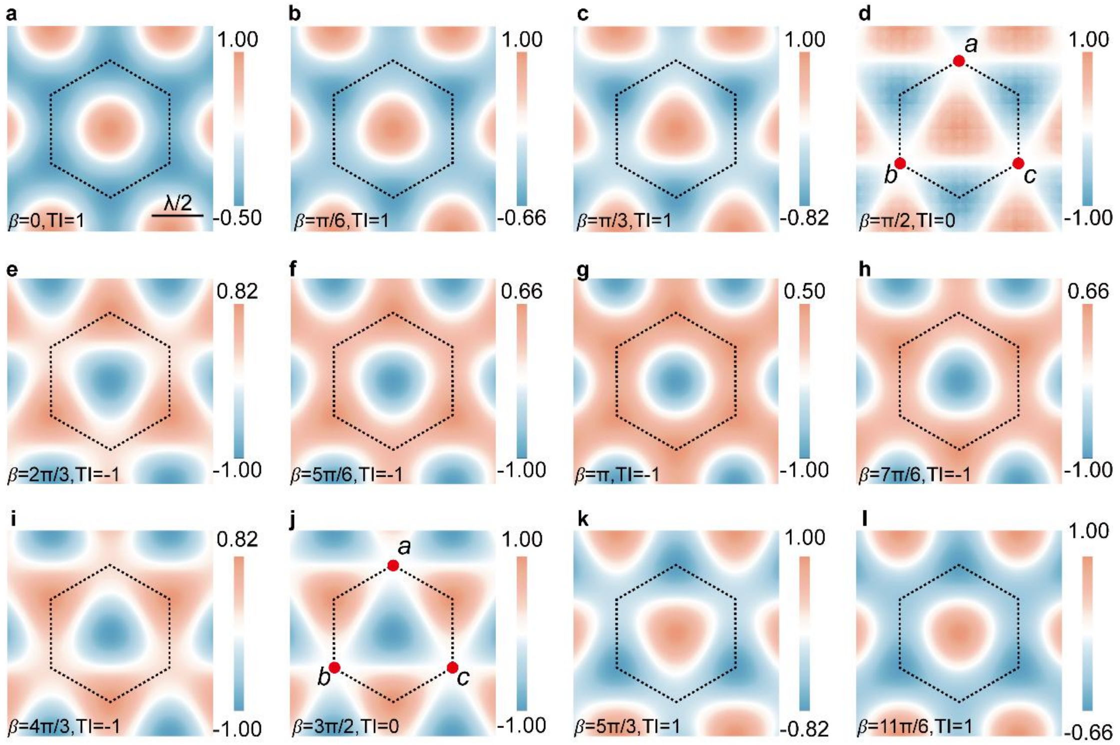

Supplementary Fig. 1| Variation of the electric field distribution described by Eq. (S3). a-l Distribution of the out-of-plane electric field component E_z when β varies from 0 (**a**) to 11π/6 (**l**) in intervals of π/6. Each field pattern is normalized by the maximum of its absolute value. The black dashed lines delineate the unit cell of the lattice. The TIs are calculated to be: 1 for (**a-c**, **k**, **l**), 0 for (**d**, **j**), and -1 for (**e-i**), respectively. The singularities in panels (**d**) and (**j**) are marked with red dots and labeled with "a", "b" and "c".

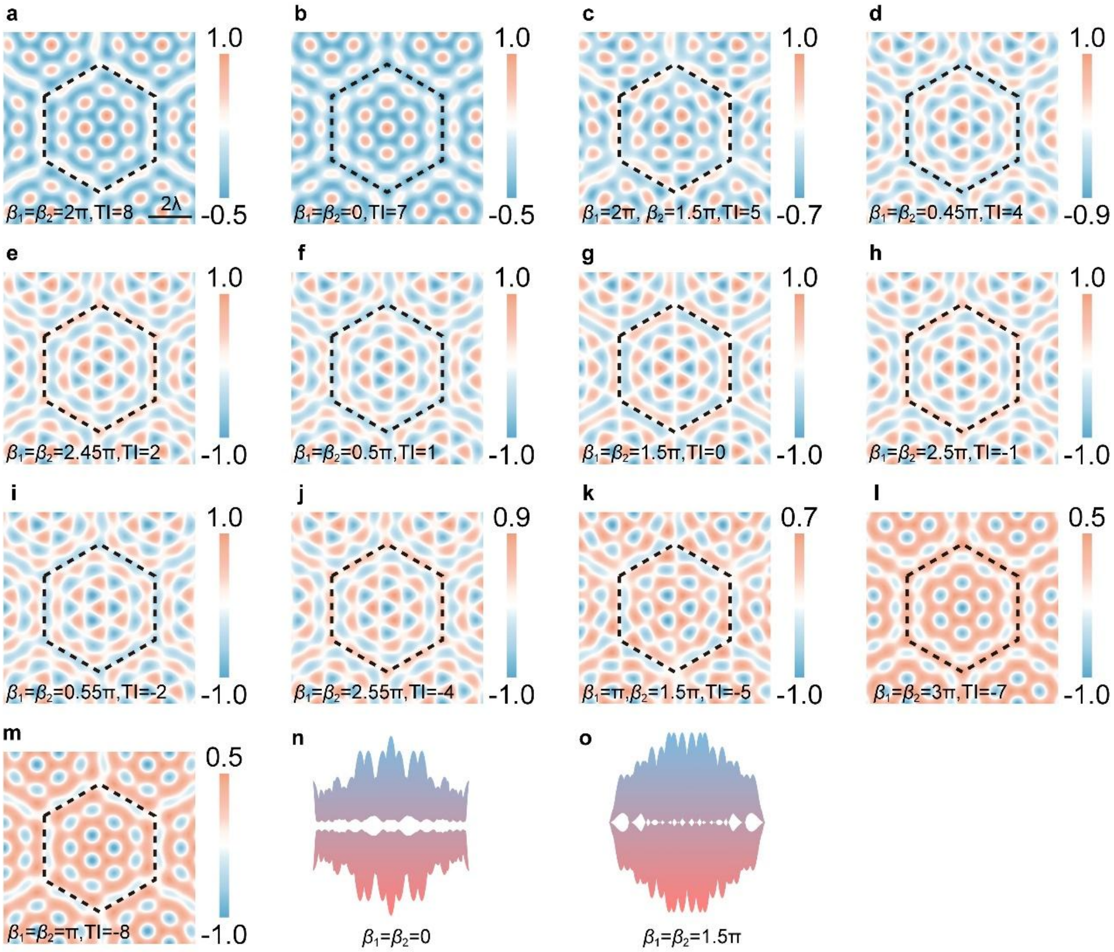

Supplementary Fig. 2| Variation of the electric field distribution (*E_z*) described by Eq. (3). a-m Distribution of the out-of-plane electric field component E_z when TIs are all integers except ±3 and ±6 within the range −8 to 8. Each field pattern is normalized by the maximum of its absolute value. The black dashed lines delineate the unit cells of the superlattices. **n**, **o** Energy band-like for β_1=β_2=0 (**n**) and β_1=β_2=1.5π (**o**).

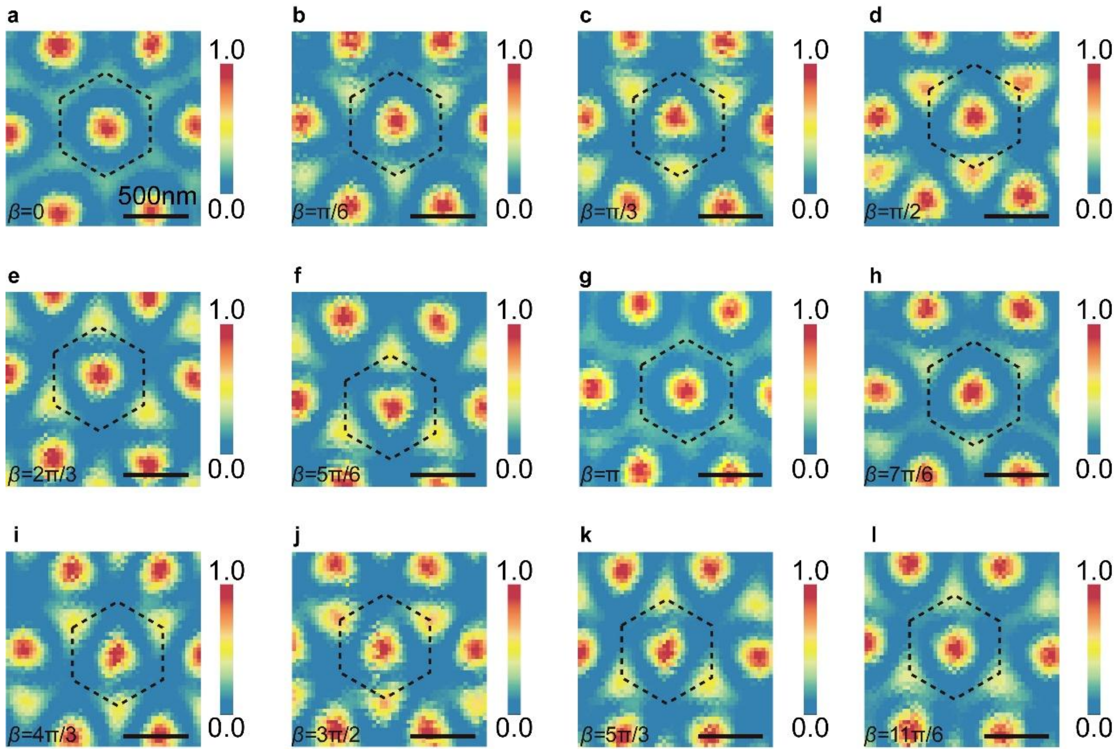

Supplementary Fig. 3| The experimentally mapped distributions of $|E_z|^2$. a-l Intensity distributions when β varies from 0 (**a**) to 11π/6 (**l**) in intervals of π/6.

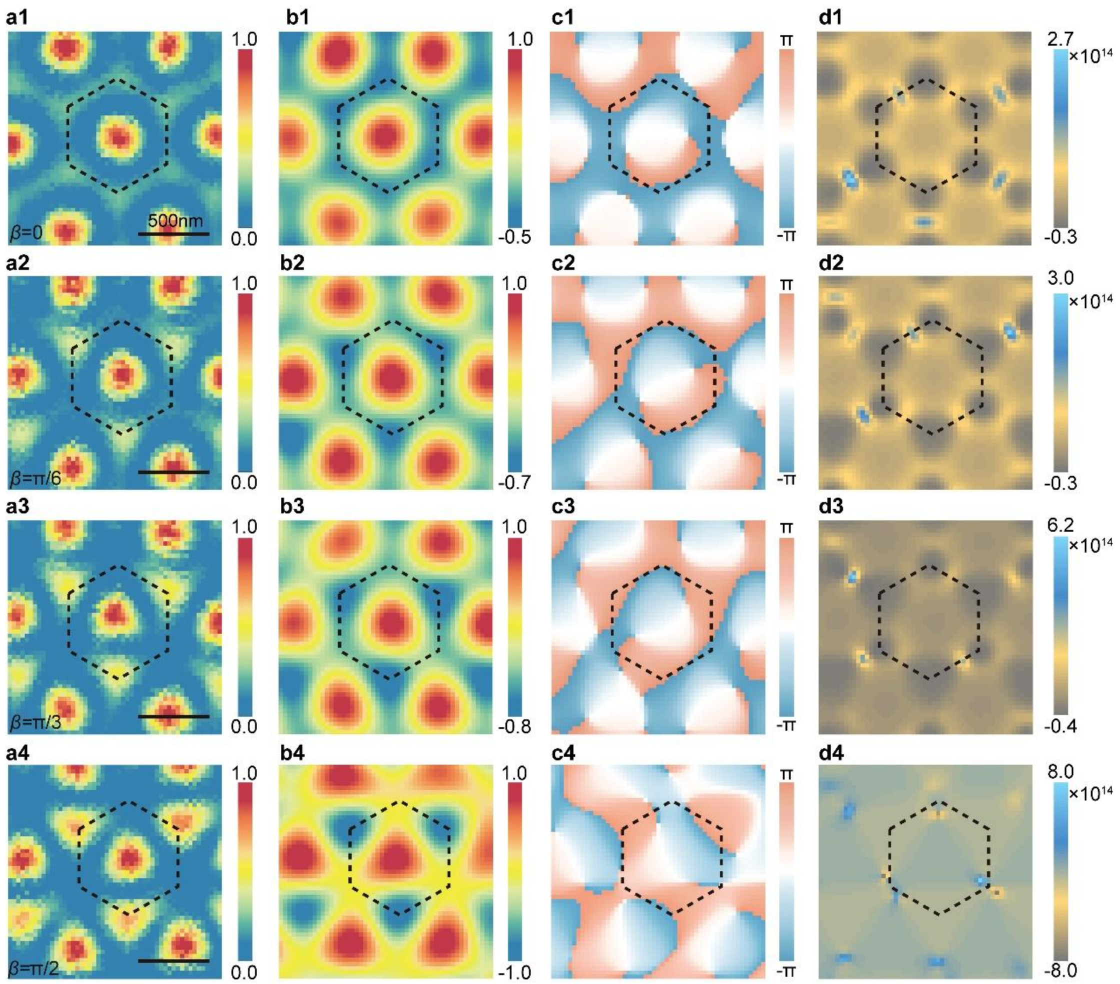

Supplementary Fig. 4| Reconstruction of the real part of E_z. a1-d4 Reconstruction of the real part (**b1-b4**) of E_z from the measured intensity distributions (**a1-a4**) shown in Supplementary Fig. 3 using the GS algorithm, where panels (**c1-c4**) are phase, panels (**d1-d4**) are topological charge density. The values of β are 0 (**a1-c1**), $\pi/6$ (**a2-c2**), $\pi/3$ (**a3-c3**), and $\pi/2$ (**a4-c4**), respectively. Although the topological charge density distributions shown in panels (**d1-d4**) are not perfectly identical to the theoretical predicted patterns, they nonetheless represent the same topological states. The topological invariant is computed through a discrete approximation of the surface integral: the topological charge density is summed over all points within a unit cell, multiplied by the area element, and normalized by 4π.

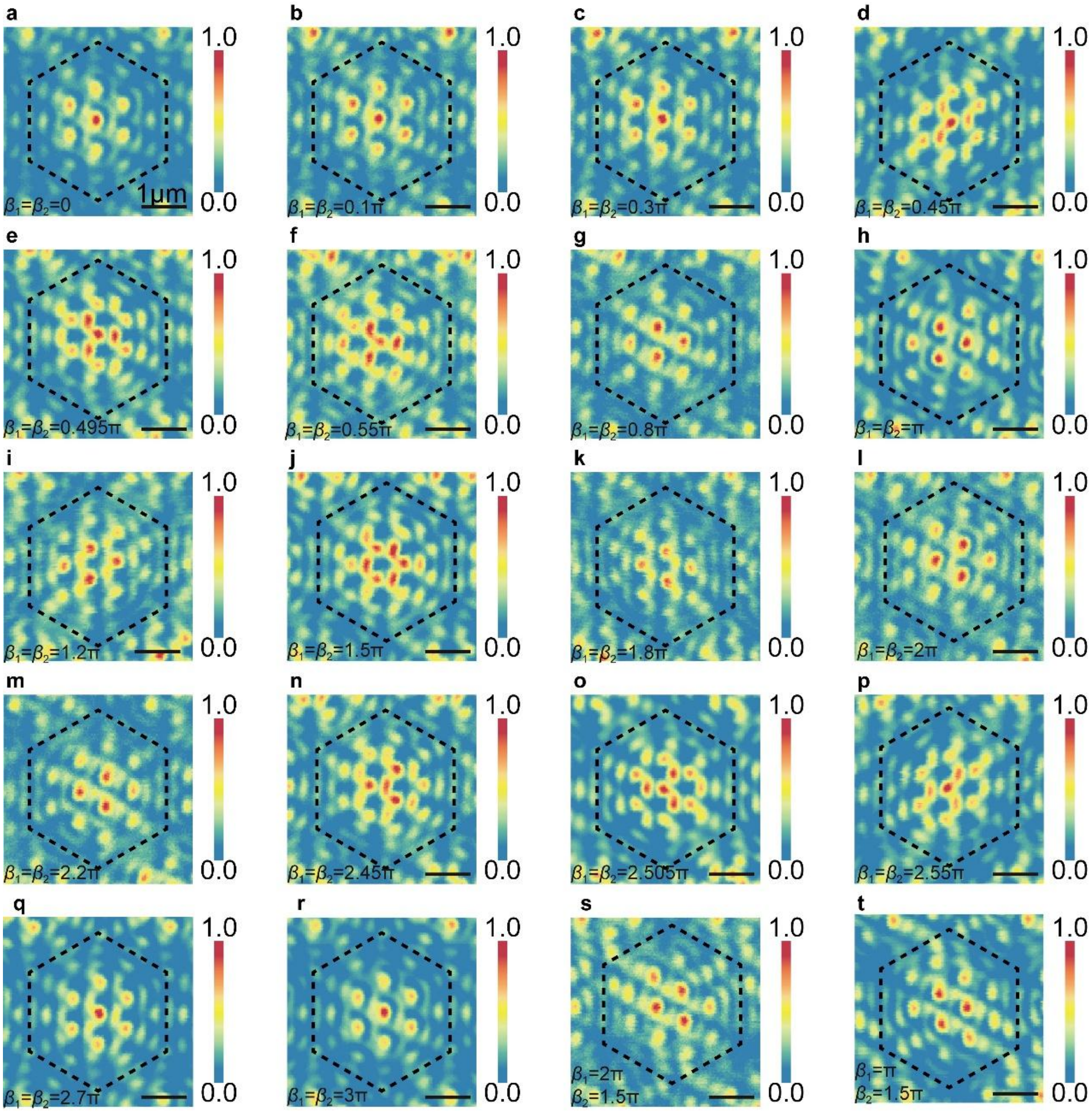

Supplementary Fig. 5| The experimentally mapped intensity distributions of $|E_z|^2$ formed by the interference of twelve TM mode evanescent beams. a-t Intensity distributions when β_1 and β_2 change independently.

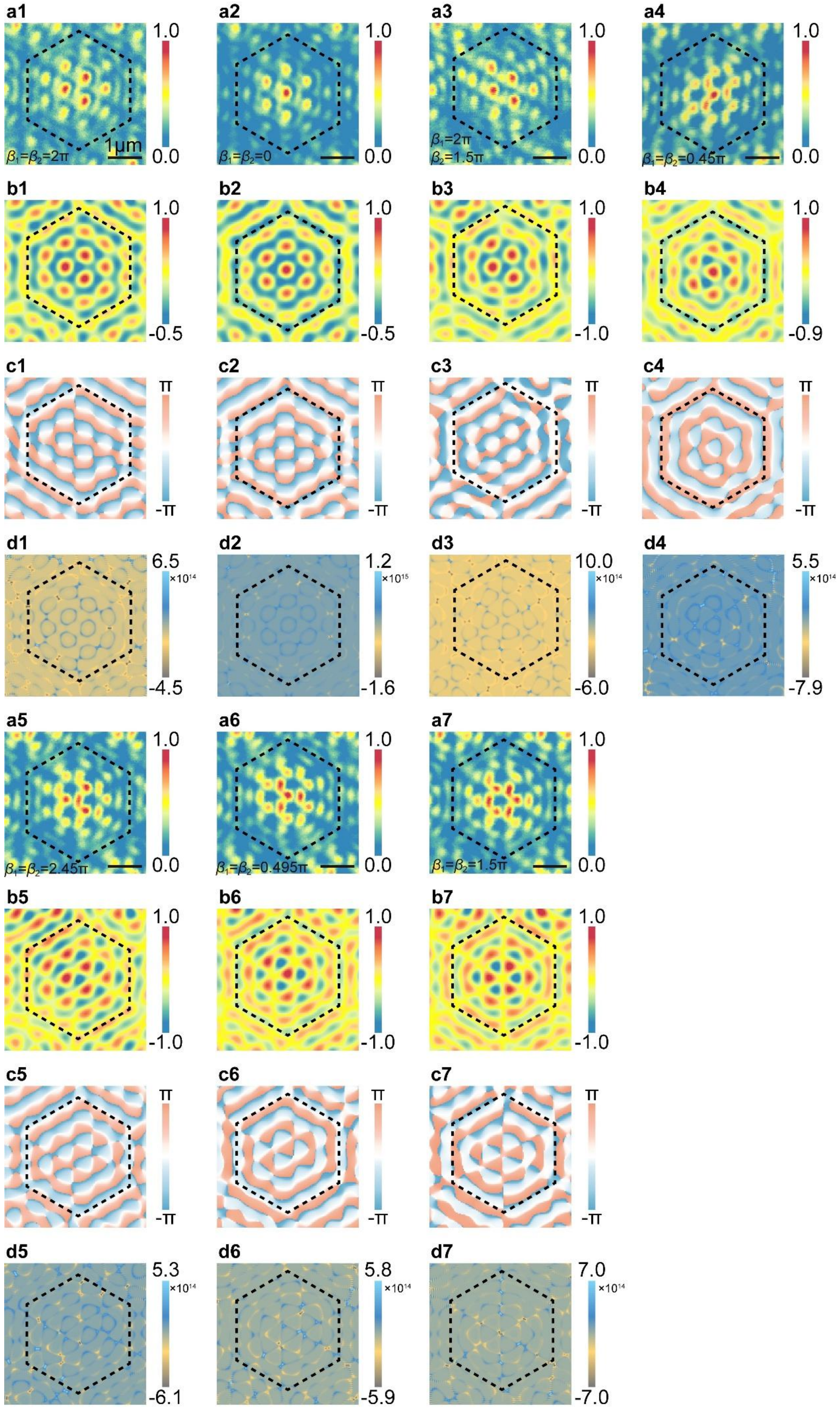

a1
1.0
0.0
1μm
$\beta_1=\beta_2=2\pi$
a2
$\beta_1=\beta_2=0$
a3
$\beta_1=2\pi$
$\beta_2=1.5\pi$
a4
$\beta_1=\beta_2=0.45\pi$
b1
-0.5
b2
b3
-1.0
b4
-0.9
c1
π
-π
c2
c3
c4
d1
6.5
×10¹⁴
-4.5
d2
1.2
×10¹⁵
-1.6
d3
10.0
-6.0
d4
5.5
-7.9
a5
$\beta_1=\beta_2=2.45\pi$
a6
$\beta_1=\beta_2=0.495\pi$
a7
$\beta_1=\beta_2=1.5\pi$
b5
b6
b7
c5
c6
c7
d5
5.3
-6.1
d6
5.8
-5.9
d7
7.0
-7.0

Supplementary Fig. 6| Reconstruction of the real part of E_z. a1-d7 Reconstruction of the real part (**b1-b7**) of E_z from the measured intensity distributions (**a1-a7**) shown in Supplementary Fig. 5 using the GS algorithm, where panels (**c1-c7**) are phase, panels (**d1-d7**) are topological charge density. Both β_1 and β_2 are 2π (**a1-c1**), 0 (**a2-c2**), 0.45π (**a4-c4**), 2.45π (**a5-c5**), 0.495π (**a6-c6**) and 1.5π (**a7-c7**), respectively. The β_1 and β_2 of (**a3-c3**) are 2π and 1.5π, respectively. Although the topological charge density distributions shown in panels (**d1-d7**) are not perfectly identical to the theoretical predicted patterns, they nonetheless represent the same topological states. The topological invariant is computed through a discrete approximation of the surface integral: the topological charge density is summed over all points within a unit cell, multiplied by the area element, and normalized by 4π.

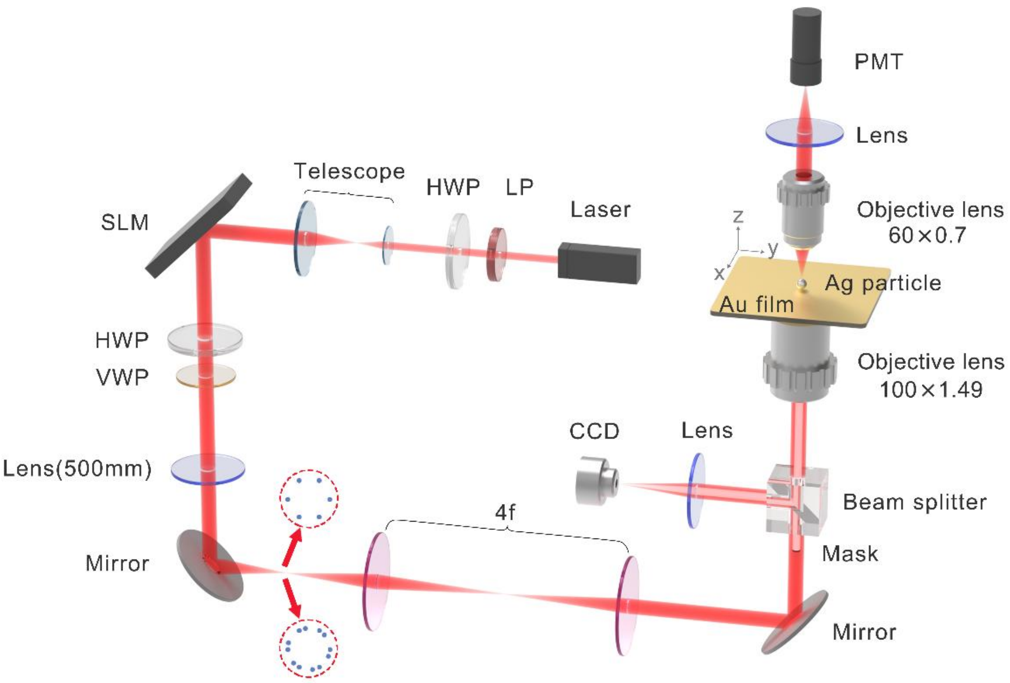

Supplementary Fig. 7| Experimental setup for characterization of topologies in TM-mode SPP. LP: linear polarizer; HWP: half-wave plate; SLM: spatial light modulator; QWP: quarter wave plate; VWP: vortex wave plate; PMT: photomultiplier tube; CCD: charge-coupled device.

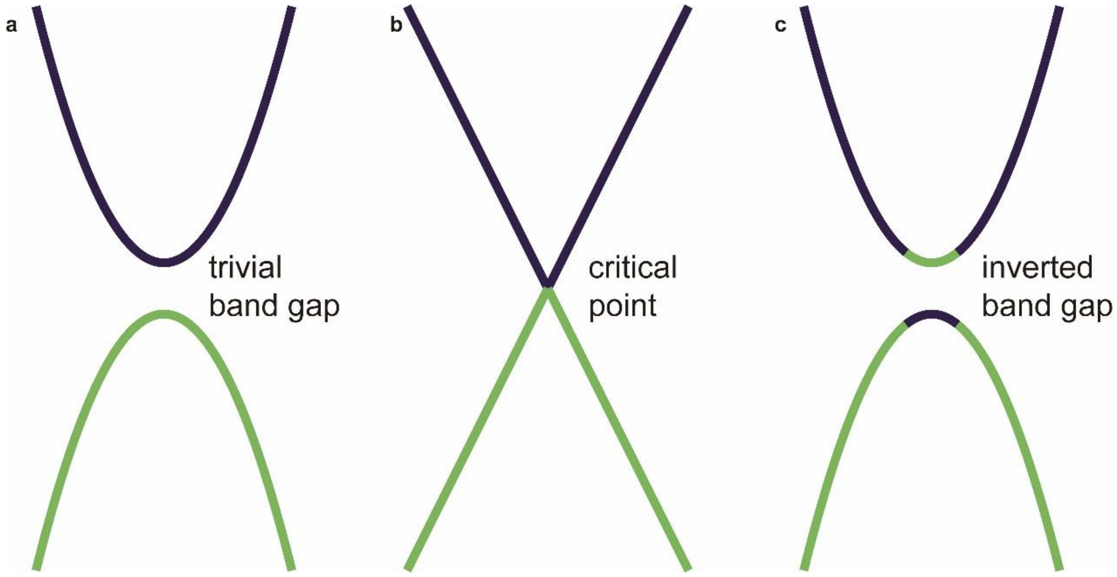

Supplementary Fig. 8| Schematic representation of the change of energy bands during a TPT. a-c Band gap (**a**) closes (**b**) at a critical point and reopens (**c**), indicating the occurrence of a topological phase transition.

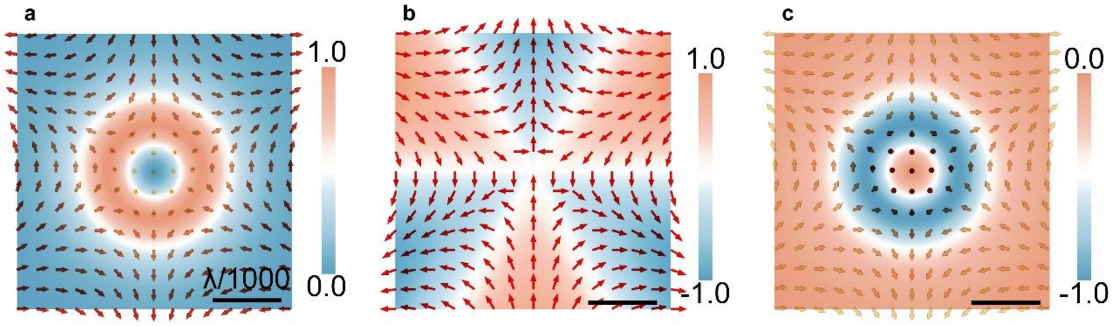

Supplementary Fig. 9| The variation of vector direction near point "a" in Supplementary Fig. 1d. a-c a singularity arises at the center of the panel (**b)** when β=π/2, leaving the vector direction undefined. Slightly deviating β from π/2 generates a bimeron with a TI of either 1(**a**) or −1(**c**) at the center. This demonstrates that as the vector field transitions from the configuration in (**a**) to (**b**) and (**c**), the TI decreases by 1 incrementally. Consequently, the TIs exhibit quantized behavior as a function of β, as shown in Fig. 1d of the main text. Notably, panels (**a)** and (**c)** represent the vector field trends for β=π/2±π/1e6; in practice, this transition occurs instantaneously as β departs from π/2.

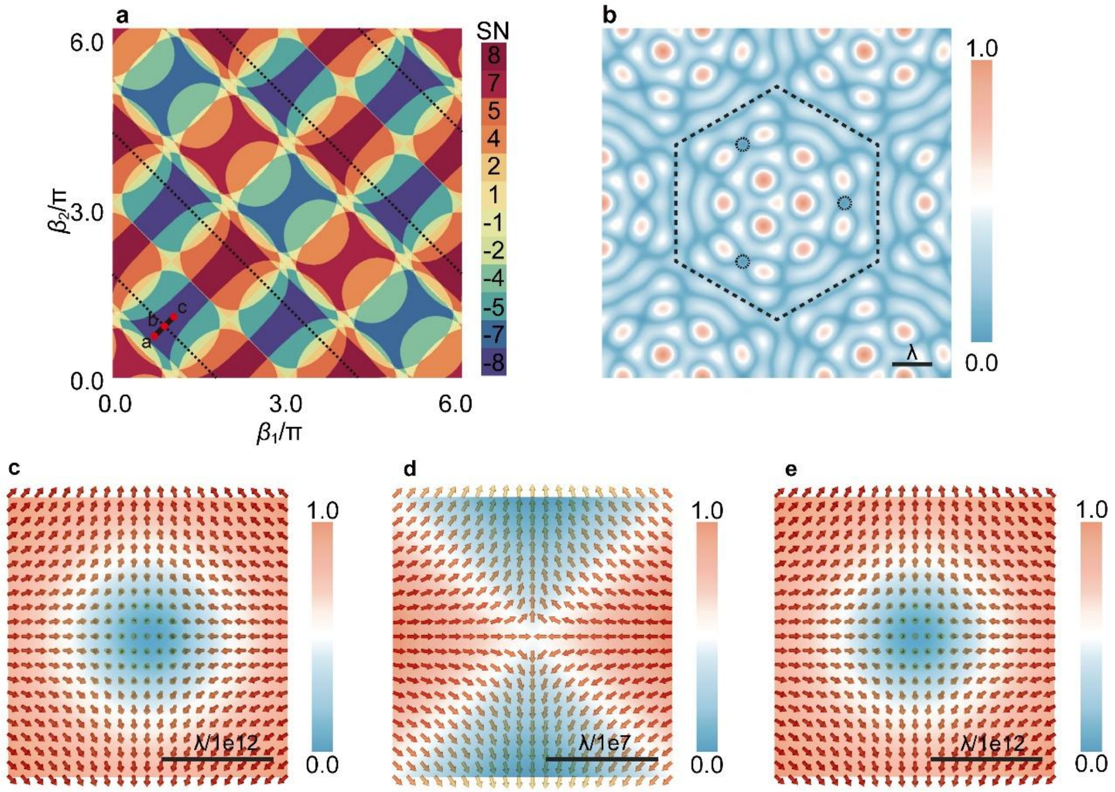

Supplementary Fig. 10| Evolution of vector fields near singularities that do not induce a topological transition. a Evolution of topological invariants as a function of β_1 and β_2. The values β_a, β_b and β_c correspond to the points labeled "a", "b" and "c", respectively. **b** The mode of the vector field described by Eqs. (S18) and (S19) for β_1=β_2=β_b. The three singularities are clearly marked by black circles. **c-e** Variation in vector orientation within the black circles of panel b for β_1=β_2=β_a (**c**), β_1=β_2=β_b (**d**), and β_1=β_2=β_c (**e**), respectively.

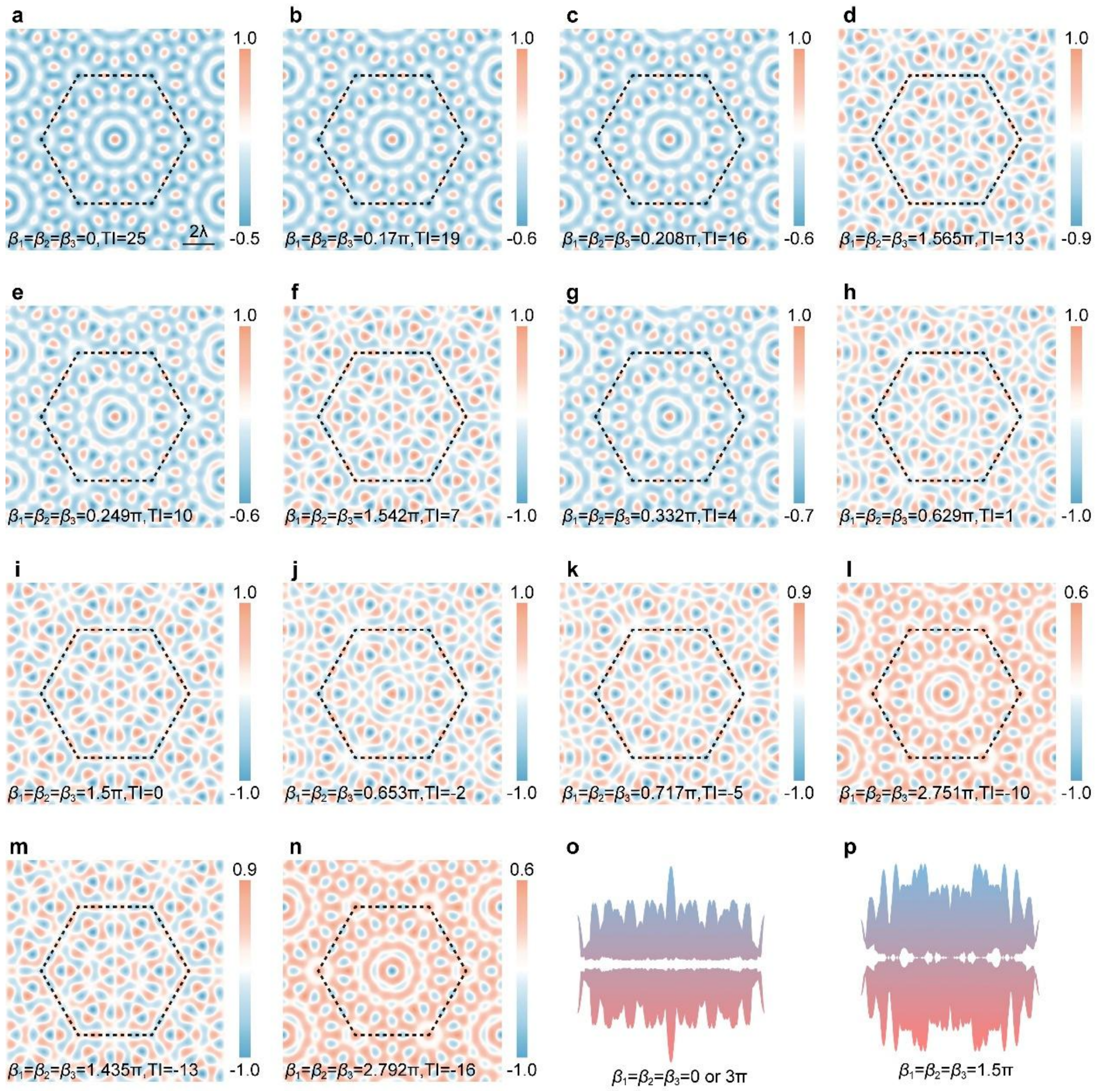

Supplementary Fig. 11| Variation of the electric field distribution (E_z) described by Eq. (S21a) and corresponding energy band-like. a-n Distribution of the out-of-plane electric field component E_z when β_1=β_2=β_3 and P=3. Each field pattern is normalized by the maximum of its absolute value. The black dashed lines delineate the unit cells of the superlattices. **o**, **p** Energy band-like for β_1=β_2=β_3=0 or 3π (**o**) and β_1=β_2=β_3=1.5π (**p**).

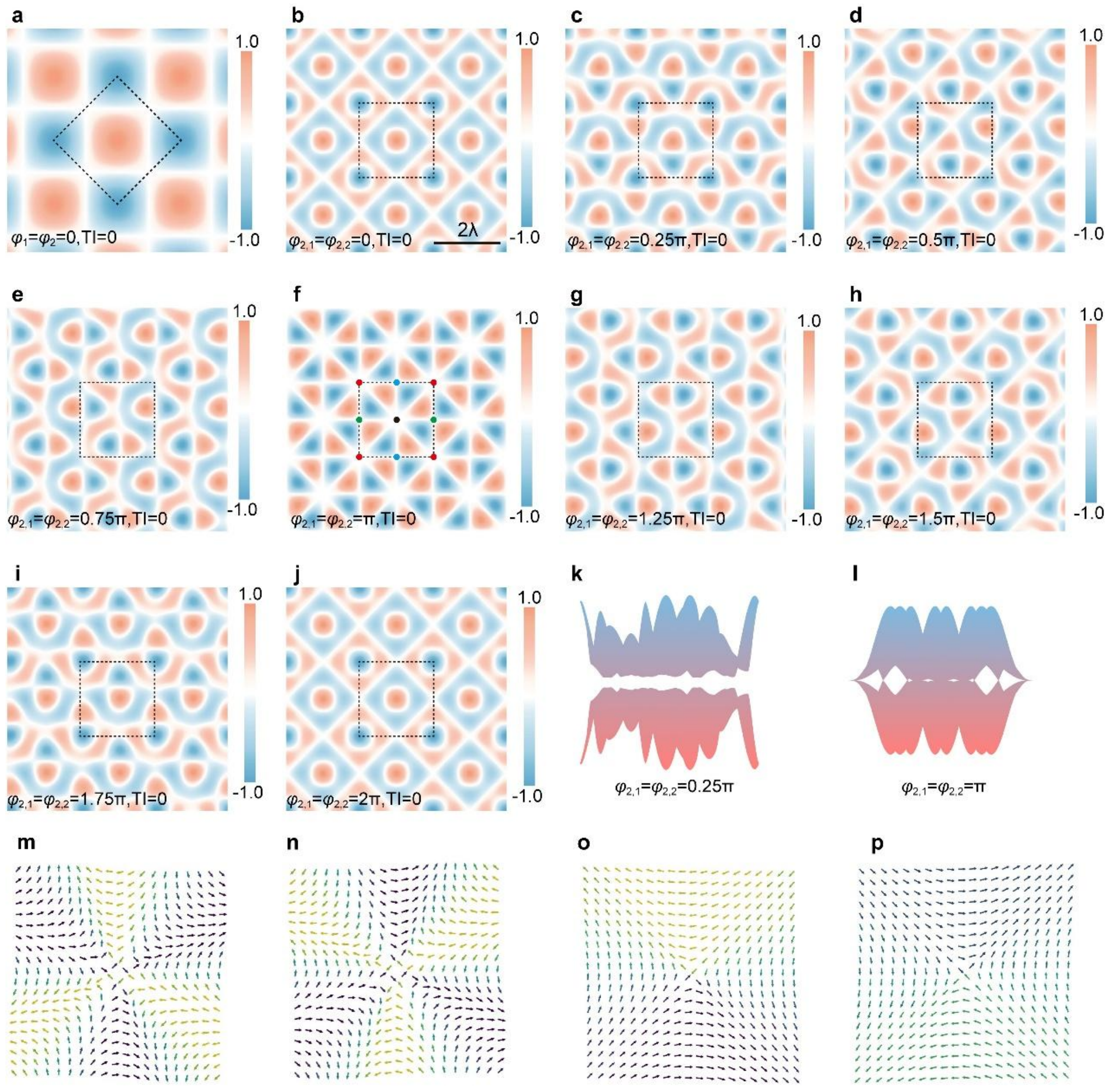

Supplementary Fig. 12| Topological states of the vector field in square Moiré superlattices. a-j Out-of-plane electric field component E_z in the square Moiré superlattice. **k, l** Energy band-like structures. **m-p** Direction of the vector field near the four types of singularities from panel (**f**).

Supplementary Table 1| Phase parameters β_1 and β_2 for different topological states at (m, n) = (3, 4).

(β_1/π, β_2/π)	TI	(β_1/π, β_2/π)	TI	(β_1/π, β_2/π)	TI
(0.269, 0.269)	19	(2.735, 2.239)	5	(2.543, 2.543)	-7
(1.872, 1.872)	17	(0.486, 0.486)	4	(0.54, 0.54)	-8
(0, 0.3)	16	(2.486, 2.486)	2	(2.83, 2.284)	-10
(2.189, 2.314)	14	(0.5, 0.5)	1	(0.599, 0.599)	-11
(0.376, 0.376)	13	(1.5, 1.5)	0	(2.624, 2.624)	-13
(2.401, 2.401)	11	(2.5, 2.5)	-1	(0.811, 0.686)	-14
(0.17, 0.716)	10	(0.517, 0.517)	-2	(3, 2.7)	-16
(2.46, 2.46)	8	(2.514, 2.514)	-4	(1.128, 1.128)	-17
(0.457, 0.457)	7	(0.265,0.761)	-5	(2.731,2.731)	-19

Supplementary Table 2| Phase parameters β_1 and β_2 for different topological states at (m, n) = (4, 9).

(β_1/π, β_2/π)	TI	(β_1/π, β_2/π)	TI	(β_1/π, β_2/π)	TI
(0, 0.1051)	58	(2.4865, 2.4865)	17	(2.534, 2.534)	-22
(0.1802, 3.9883)	56	(0.4779, 0.4779)	16	(0.5232, 0.5232)	-23
(0, 0)	55	(2.695, 2.297)	14	(2.4395, 2.6597)	-25
(2.1091, 2.1091)	53	(0.4837, 0.4837)	13	(0.6756, 0.3703)	-26
(0.085, 0.155)	52	(2.4932, 2.4932)	11	(2.556, 2.556)	-28
(2.318, 2.318)	50	(0.4895, 0.4895)	10	(0.5381, 0.5381)	-29
(0.0997, 0.0997)	49	(2.4981, 2.4981)	8	(2.2594, 2.8299)	-31
(2.0362, 2.0362)	47	(0.1952, 0.7907)	7	(0.555, 0.555)	-32
(0.3467, 0.3467)	46	(2.4995, 2.4995)	5	(2.567, 2.567)	-34
(2.3603, 2.3603)	44	(0.4938, 0.4938)	4	(2.3845, 0.7431)	-35
(0.205, 0.565)	43	(2.5017, 2.5017)	2	(2.685, 2.505)	-37
(1.7055, 1.7055)	41	(0.1902, 0.8057)	1	(0.5788, 0.5788)	-38
(0.4017, 0.4017)	40	(1.5, 1.5)	0	(2.5983, 2.5983)	-40
(2.4212, 2.4212)	38	(2.8098, 2.1943)	-1	(1.2945, 1.2945)	-41
(0.315, 0.495)	37	(0.4983, 0.4983)	-2	(2.795, 2.795)	-43
(0.6155, 2.2569)	35	(2.5062, 2.5062)	-4	(0.6397, 0.6397)	-44
(0.433, 0.433)	34	(0.5005, 0.5005)	-5	(2.6533, 2.6533)	-46
(2.445, 2.445)	32	(2.8048, 2.2093)	-7	(0.9638, 0.9638)	-47
(0.7406, 0.1701)	31	(0.5019, 0.5019)	-8	(2.9003, 2.9003)	-49
(2.4619, 2.4619)	29	(2.5105, 2.5105)	-10	(0.682, 0.682)	-50
(0.444, 0.444)	28	(0.5068, 0.5068)	-11	(2.915, 2.845)	-52
(2.3244, 2.6297)	26	(2.5163, 2.5163)	-13	(0.8909, 0.8909)	-53
(0.5605, 0.3403)	25	(0.305, 0.703)	-14	(3, 3)	-55
(2.4768, 2.4768)	23	(2.5221, 2.5221)	-16	(2.8198, 5.0117)	-56
(0.466, 0.466)	22	(0.5135, 0.5135)	-17	(3, 3.1051)	-58
(1.4787, 2.4145)	20	(2.99, 2.11)	-19		
(0.01, 0.89)	19	(1.5213, 0.5855)	-20		

Supplementary References

1 Maier, S. A. *Plasmonics: Fundamentals and Applications* (Springer, 2007).

2 Hasan, M. Z. & Kane, C. L. Colloquium: Topological insulators. *Rev. Mod. Phys.* **82**, 3045–3067 (2010).

3 Shen, S.-Q. *Topological Insulator* (Springer, Berlin, 2012).

4 Tsesses, S. et al. Optical skyrmion lattice in evanescent electromagnetic fields. *Science* **361**, 993–996 (2018).
